# Adélie penguins north and east of the ‘Adélie gap’ continue to thrive in the face of dramatic declines elsewhere in the Antarctic Peninsula region

**DOI:** 10.1038/s41598-023-29465-4

**Published:** 2023-02-13

**Authors:** Michael Wethington, Clare Flynn, Alex Borowicz, Heather J. Lynch

**Affiliations:** 1 Department of Ecology and Evolution, Stony Brook University, Stony Brook, NY 11794 USA; 2 School of Marine and Atmospheric Sciences, Stony Brook University, Stony Brook, NY 11794 USA; 3 Institute for Advanced Computational Science, Stony Brook University, Stony Brook, NY 11794 USA

**Keywords:** Population dynamics, Cryospheric science, Ecology

## Abstract

While population declines among Adélie penguins and population increases among gentoo penguins on the Western Antarctic Peninsula are well established, the logistical challenges of operating in the sea ice-heavy northern tip of the Antarctic Peninsula have prohibited reliable monitoring of seabirds in this region. Here we describe the findings of an expedition to the northern and eastern sides of the Antarctic Peninsula—a region at the nexus of two proposed Marine Protected Areas—to investigate the distribution and abundance of penguins in this region. We discovered several previously undocumented penguin colonies, completed direct surveys of three colonies initially discovered in satellite imagery, and re-surveyed several colonies last surveyed more than a decade ago. Whereas our expectation had been that the Peninsula itself would divide the areas undergoing ecological transition and the apparently more stable Weddell Sea region, our findings suggest that the actual transition zone lies in the so-called "Adélie gap," a 400-km stretch of coastline in which Adélies are notably absent. Our findings suggest that the region north and east of this gap represents a distinct ecoregion whose dynamics stand in sharp contrast to surrounding areas and is likely to be impacted by future conservation measures.

## Introduction

**Figure 1:**
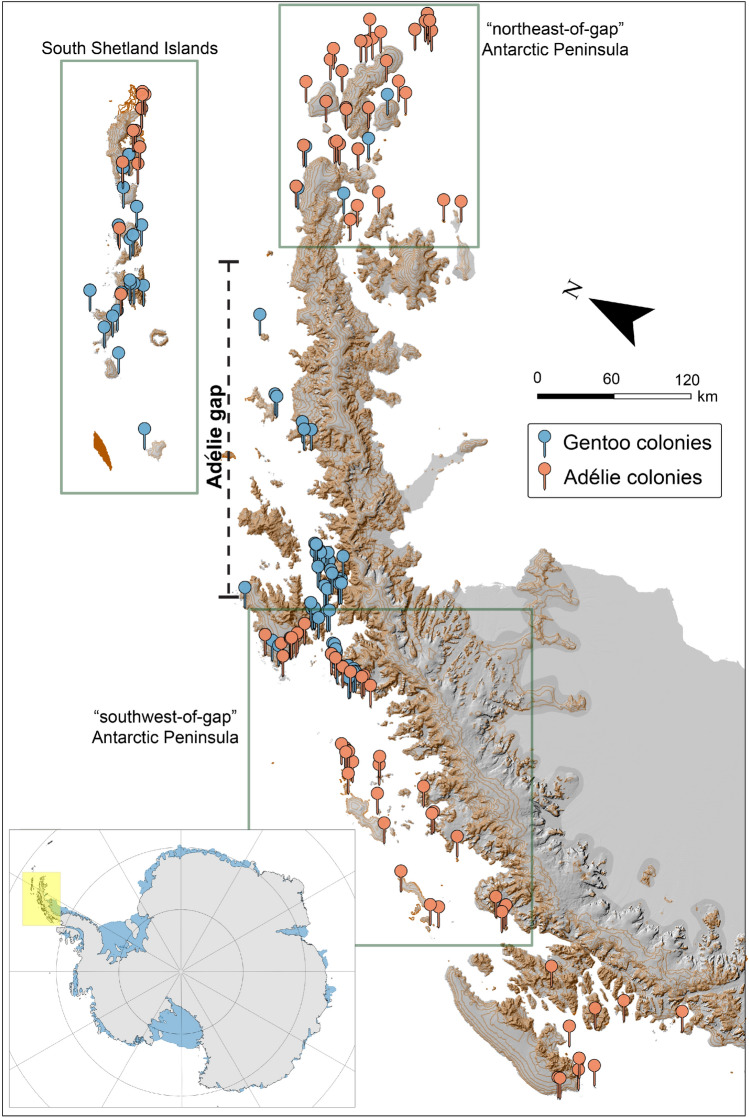
Adélie and gentoo penguin colonies on the Antarctic Peninsula. Lower left inset: Antarctic Peninsula region (yellow) in the context of the entire Antarctic continent. Figure created using ArcGIS Pro 3.0 (https://pro.arcgis.com).

**Figure 2:**
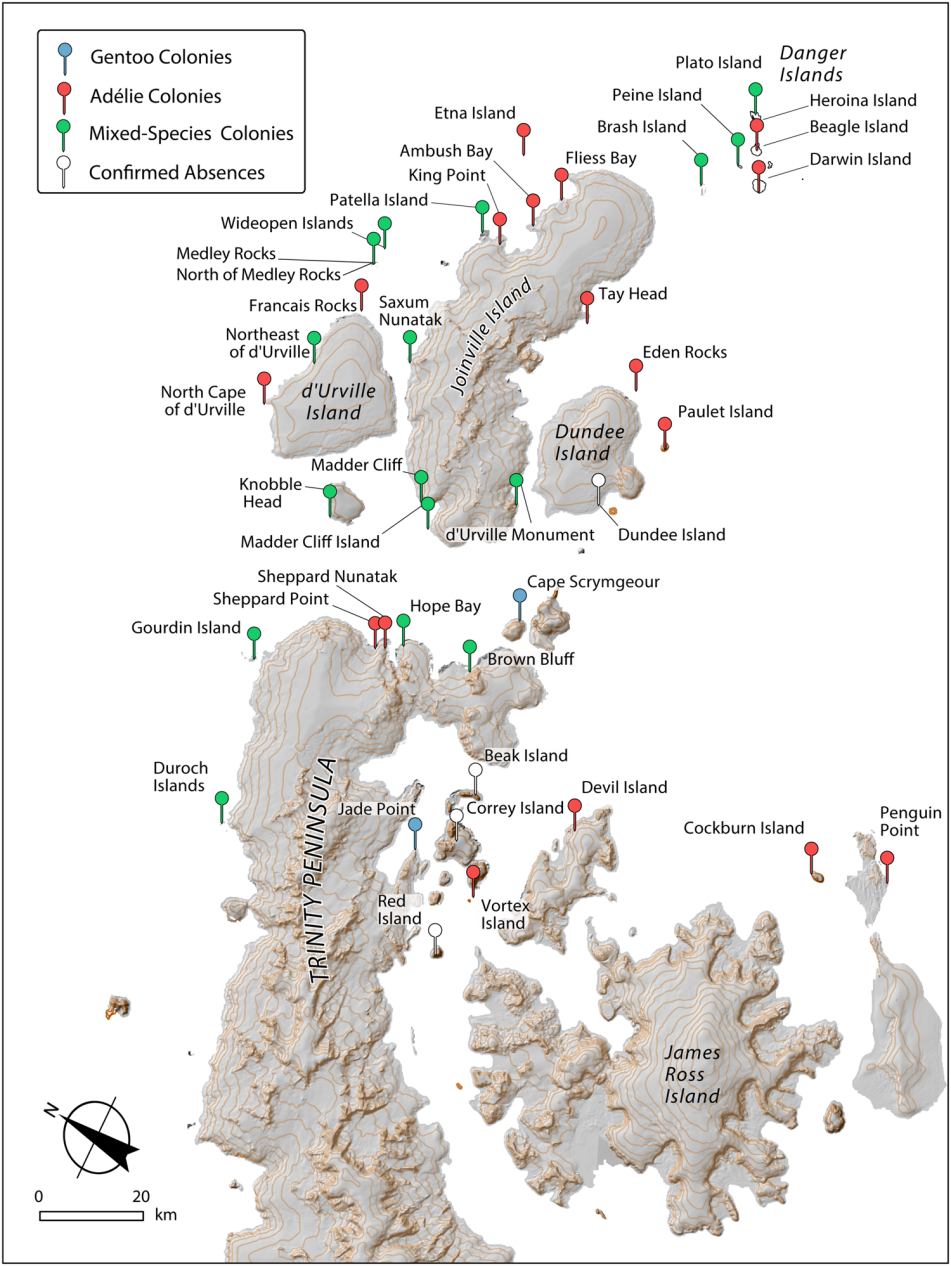
Adélie and gentoo penguin colonies located in the ’Northeast-of-gap’ region. Figure created using ESRI ArcGIS Pro 3.0 (https://pro.arcgis.com).

Over the last 40+ years, there have been a series of hypotheses put forth to explain the dramatic changes observed among brush-tailed penguins breeding on the Western Antarctic Peninsula (WAP)^1–4^, where relatively easy access by yachts and cruise ships and the presence of several major research stations has facilitated the monitoring of penguin populations since the late 1970s. One of the notable features of penguin range distributions in this region is the complete absence of breeding Adélie penguins over a 400-km stretch extending between Anvers Island in the south and King George Island and the Duroch Islands on the west coast of Trinity Peninsula—a feature known as the "Adélie gap"^1,5^ (Fig. 1). The existence of the Adélie gap has led several researchers to hypothesize the existence of two distinct populations for this species^1,6^, such that Adélie penguins breeding north of the Adélie gap, alongside those breeding in the South Shetland and South Orkney Islands, were tied to the icy conditions of the Weddell Sea, unlike conspecifics breeding south of the Adélie gap that were reliant instead on the Bellinghausen Sea. This observation that Adélie penguins are absent in the so-called Adélie gap (Fig. 1) has been confirmed^7^, alongside compelling data showing how Adélie penguins south of the gap have declined sharply over the last several decades^8^. Far less is known about the populations north of the Adélie gap along the northern tip of the Antarctic Peninsula and island groups east of the Peninsula in the northern fringes of the Weddell Sea (Fig. 2). Biologically-focused expeditions to this region have been remarkably sparse, and most of the data available from the 20th century derive from a pair of geological expeditions in which the presence of penguins was noted only in passing^9^. A few colonies in this region, such as those at Devil Island, Paulet Island, and Brown Bluff, are regularly visited by commercial cruise vessels^10^, and these provide the only consistent time series available for penguin colonies in the region^7^. More recently, Earth-orbiting satellites have been used to document the presence of penguin guano in the remaining poorly-surveyed regions and to estimate abundance based on the geographic area of the guano stain created at the breeding site^11,12^, but direct ground census work has remained extremely limited.

Waters off the WAP have experienced notable declines in annual sea-ice extent over the past several decades^13,14^. In contrast, in the Weddell Sea, overall sea-ice extent showed moderate increases between 1979 and 2015, before more recent and sudden decreases beginning in 2016^15–17^. Kumar et al.^16^ found that a reduced sea ice extent year in 2015/16 closely followed a strong El Nino Southern Oscillation (ENSO) event, an irregular periodic variation in winds and sea surface temperatures over the tropical eastern Pacific Ocean that affects the climate of much of the tropics and subtropics. Coincident with the ENSO, a positive phase of the Southern Annual Mode co-occurred that same year, causing major ice loss and potentially shifting the Weddell Sea into an alternative state of sea-ice decline^16^. Moreover, the Weddell Sea gyre may be perpetuating a stabilizing negative feedback loop, where a decrease in sea ice leads to a strengthening of the gyre, resulting in surface temperatures that support higher sea-ice concentration^18^. Under the assumption of relative stability in annual sea-ice extent, the Weddell Sea has been projected to have increasingly suitable habitat for Antarctic krill, while the WAP is expected to have an increase in habitat in the spring, but a decline in the summer and fall^19^.

An ongoing question is the extent to which differences in habitat and climate patterns along the Eastern Antarctic Peninsula differ relative to the WAP. While it is well established that Adélie penguin populations along the WAP and sub-Antarctic islands, have declined sharply over the last several decades^20^, trends remain less well-understood among the eastern populations in the Weddell Sea region, the northeastern-most area of their breeding range along the Peninsula. Several years ago, satellite-based observations of penguin guano in the Danger Islands archipelago, near the eastern tip of Joinville Island, motivated a major expedition to the region^21^. In addition to finding several large Adélie colonies not previously documented, the expedition discovered that 55% of all Adélie penguins in the Antarctic Peninsula region nest in the Danger Islands, making it an unexpected hotspot of Adélie abundance in a region of Antarctica projected to remain favorable for Adélie reproduction even under forecasted climate change scenarios^22^. Importantly, Borowicz et al.^21^ compared recent high-resolution satellite imagery with older Landsat imagery and historic aerial photography, finding evidence that Adélie populations in the Danger Islands appeared broadly stable over the last 60 years, in marked contrast to the long-reported Adélie population declines on the WAP^8,23^, and in areas of the South Shetland^2,24^ and South Orkney Islands^25^. This apparent dichotomy between the trends of Adèlie penguins on the east and west sides of the Antarctic Peninsula, alongside the effort to establish two Marine Protected Areas in the region^26–28^, motivated an expedition in the 2021/22 austral summer to the lesser-surveyed areas of the northern Peninsula and Weddell Sea.

In contrast to patterns of Adélie penguin population decline throughout much of the Antarctic Peninsula, gentoo penguins (*Pygoscelis papua*), a historically sub-antarctic species of *Pygoscelis* penguin, have shown consistent southward range expansions along the Peninsula and concurrent increases in population size^8,29,30^. While previous work has shown that Adélie penguins rely on areas that experience extensive annual sea ice, an important over-wintering habitat for their primary dietary staple (krill), gentoo penguins are known for greater flexibility in diet composition and foraging behaviour^31,32^, and occupy regions characterized by reduced ice-cover^8,33^. As a resident species that remains in the vicinity of the breeding colony year round, gentoos are particularly sensitive to winter sea ice conditions, as extensive winter sea ice coverage near their breeding colony could preclude easy access to the ocean required for winter foraging^8^. So, while declining sea-ice extent around the WAP may be beneficial for gentoo penguin expansion, the relatively icy environment of the Weddell Sea, particularly during the overwintering months, may be less hospitable.

Past observations that the Weddell Sea climate may be changing less rapidly than its western counterpart^13^, in addition to the recent discovery of several large and apparently stable Adélie penguin colonies in the Danger Islands vicinity, led us to search for a geographic boundary dividing the declining Adélie penguin populations of the WAP from stable colonies off the tip of the Antarctic Peninsula and into the Weddell Sea. This expedition set out to determine whether infrequently visited Adélie colonies in the Weddell Sea have indeed remained stable since their last survey, and whether gentoo penguins have also expanded into the icier Weddell Sea. Finally, we assess the multi-decadal trends of sea-ice concentration across the Antarctic Peninsula, specifically looking at trends north and south of the Adélie gap. While broad trends in pygoscelid abundance have driven much of the debate over the last 40+ years, a closer examination of ecological boundaries within the Antarctic Peninsula region may inform our understanding of the mechanistic causes underlying the observed changes in distribution and abundance.

## Methods

Field-based surveys of Adélie and gentoo penguin colonies were conducted from the Motor Vessel Arctic Sunrise from January 15 to February 4, 2022 (Fig. 2, Table 1). We used two different survey methods depending on landing accessibility, environmental conditions, and available time ashore: (1) manually hand-counting individual penguin chicks, and (2) remotely—piloted aircraft system (RPAS) surveys yielding aerial photographic mosaics of the colony from which counts of individuals could be derived through manual annotation using a Geographic Information System. Because the timing of the expedition occurred during the latter half of the breeding season^34^, an unavoidable consequence of the Covid-19 pandemic, we surveyed chicks rather than nests. Both Adélie and gentoo penguin colonies had 3- to 4+ week-old chicks and in several cases had already créched (but not fledged) prior to our arrival. Penguin chick fraction was estimated using portions of the RPAS captured scenes at each site where chicks could be unambiguously identified (see example image in Supplementary Materials A). Because surveys were completed on different days and chick fraction is sensitive to breeding phenology, we estimated each site’s chick fraction separately rather than pooling the estimates. All observers involved in this study were contributing authors.

**Table 1:** Available Adélie penguin census data for colony locations surveyed during this expedition.

Site	Year	Count	Citation	Method
Cockburn Island (COCK)	2011	15721 (N5)	^12^	vhr
	2021	4274 (C)	This survey	RPAS
Devil Island (DEVI)	1996	10320 (C2)	^67^	Ground
	2000	8501 (C2)	^67^	Ground
	2000	6365 (C4)	^67^	Ground
	2002	5880 (C3)	^67^	Ground
	2003	8500 (C3)	^67^	Ground
	2004	8802 (N2)	^67^	Ground
	2007	18000 (C3)	^67^	Ground
	2009	20040 (C1)	^67^	Ground photo
	2008	14681 (N1)	^67^	Ground photo
	2011	7108 (C1)	^67^	Ground
	2021	11978 (C1)	This survey	Ground/RPAS
Eden Rocks (EDEN)	1996	46855 (N3)	^67^	Ground
	2011	45374 (N5)	^12^	vhr
	2021	43709 (C)	This survey	RPAS
Medley Rocks 1 (part of MEDL)	2021	238 (C1)	This survey	Ground
Medley Rocks 2 (part of MEDL)	2021	312 (C1)	This survey	Ground
Medley Rocks 3 (part of MEDL)	2021	125 (C1)	This survey	Ground
Medley Rocks 4 (part of MEDL)	2021	1955 (C1)	This survey	Ground
Medley Rocks 5 (part of MEDL)	2021	733 (C1)	This survey	Ground
Medley Rocks 6 (part of MEDL)	2021	611 (C1)	This survey	Ground
Medley Rocks 7 (part of MEDL)	2021	528 (C1)	This survey	Ground
Medley Rocks 8 (part of MEDL)	2021	168 (C1)	This survey	Ground
Medley Rocks 9 (part of MEDL)	2021	70 (C1)	This survey	Ground
N. of Medley Rocks (NMED)	2021	1008 (C1)	This survey	Ground
Penguin Point (PEPO)	1985	21954 (N1)	^68^	Ground
	2000	20061 (C3)	^69^	Ground/mas
	2006	26400 (N4)	^67^	Ground
	2009	16015 (N2)	^67^	Ground
	2014	29069 (N1)	^70^	Ground
	2021	21561 (C1)	This survey	Ground/RPAS
Northeast of d’Urville (NEDU)	2021	1253 (C1)	This survey	Ground/RPAS
Tay Head (TAYH)	2002	17500 (N4)	^67^	Ground
	2006	6450 (N4)	^67^	Ground
	2010	4156 (N3)	^67^	Ground
	2021	6769 (C1)	This survey	Ground
Vortex Island (VORT)	2008	4319 (N2)	^67^	Ground
	2011	2665 (N5)	^12^	vhr
	2021	6937 (C1)	This survey	Ground
West of Tay Head (WTAY)	2021	3000 (N5)	This survey	Vessel

Additional data from previous surveys have been included so as to place new data in its historical context; these data may also be downloaded by the Mapping Application for Penguin Populations and Projected Dynamics^7^ (MAPPPD; www.penguinmap.com). Each site’s 4 letter MAPPPD code has been included. Ground, Survey was conducted by researchers on the ground; RPAS, Remotely-piloted aircraft systems; vhr, very-high resolution commercial satellite imagery; vessel, survey conducted by an offshore vessel.

All research was conducted with under the approval of Stony Brook University’s Institutional Animal Care and Use Committee (237420). This expedition was permitted under Antarctic Conservation Act Permit ACA 2022-017 and ACA 2022-018. An Initial Environmental Evaluation was approved by the US Environmental Protection Agency on 14 October 2021, and Advance Notification provided to the US Department of State.

### Manual surveys

Manual ground counts were conducted by experienced observers following previously-established methods and procedures^24^. Wherever possible, observers maintained a minimum of 5 m distance from the penguin colony to minimize disturbance of wildlife. Colonies were surveyed by direct counting of penguin chicks using handheld tally counters. To ensure count accuracy, penguin chicks were counted individually three times, with the requirement of counts being within 5% of their mean. Using this method, the mean number of chicks was assumed to be accurate to ± 5%, the accuracy threshold corresponding to the ’N1’ level of precision described by Croxall and Kirkwood^35^ and used regularly to report penguin abundance in the Antarctic^36–38^.

### Unmanned aerial surveys

RPAS surveys were performed using a DJI Phantom 4 Pro V2 (DJI, Shenzen, China) quadcopter using the stock CMOS FC6310S camera. Depending on conditions, the RPAS was flown either manually or automatically using DJI Ground Station Pro. At each survey site, weather permitting, a RPAS team consisting of a Federal Avaiation Administration licensed remote pilot and an observer conducted RPAS survey operations. While the pilot operated the RPAS, the observer maintained visual contact with the RPAS at all times, in accordance with US FAA regulations. At one location, challenging terrain precluded landing and executing a ground-based RPAS survey. In this case, the RPAS was deployed from a rigid-hulled inflatable boat. In all other locations, surveys were conducted on land within the vicinity of the colony.

RPAS-captured images were collected along flight transects using the autonomous flight planning software DJI Ground station Pro (https://www.dji.com/ground-station-pro). Images were captured every 2 s (equal time interval mode), with a constant velocity of 3 m/s (low speed), to minimize motion blur and to ensure sharp and correctly-exposed images. Flight heights were determined by the geography, take off locations, and ruggedness of the terrain but were no less than 30 m above the colony. No disturbance to nesting penguins was observed during flight operations. Image collection parameters were set to obtain image overlap of 70% in both front and side directions to ensure reliable alignment of images and to reduce the distortions in the resulting orthomosaic data products^39^. After completing each RPAS survey, RPAS-captured images were processed on Agisoft PhotoScan Professional (Agisoft LLC, St Petersburg, Russia, http://www.agisoft.com/) commercial photogrammetry software using structure-from-motion processing^40^.

To determine the total number of penguin chicks in RPAS image orthomosaics, individual penguins were marked and counted by trained remote-sensing analysts using GIS software. Each penguin within the study area colony was delineated by appending a spatial data point to its location using ArcGIS Pro (Version 2.8). At two sites, Cockburn Island and Eden Rocks, image clarity was insufficient to separate adults from chicks in some areas. To circumvent this issue, analysts counted every penguin at each colony regardless of age class, then selected a sample of high quality image sections from each colony and differentiated between penguin adults and chicks to obtain the ratios of chicks to adults. The ratios were then applied across each colony to estimate the total number of chicks at each site. This process was also employed for Devil Island, a colony where accurate hand counts were obtained the same day as the RPAS survey. This allowed us to compare our extrapolation method to the more traditional method of hand counting chicks from the ground.

### Statistical analysis of multi-decadal quarterly trends of sea-ice concentration

To investigate patterns of change in sea-ice concentration across regions of the Antarctic Peninsula through time (1979–2021) data, we used 25 km × 25 km resolution gridded monthly sea-ice concentration (the fraction of ocean surface covered by ice) product obtained from satellite-based passive-microwave sensors. The data were processed by the NASA Team algorithm^41^ and acquired via the US National Snow and Ice Data Center^42^. Sea-ice concentration products used in this study were derived from two data sets: The Near-Real-Time Defense Meteorological Satellite Program (DMSP) Special Sensor Microwave Imagery Sounder (SSMIS) Daily Polar Gridded sea-ice concentrations and the sea-ice concentrations from Nimbus-7 Scanning Multichannel Microwave Radiometer Passive Microwave data. A 25 km × 25 km land mask was applied to all sea-ice concentration rasters to eliminate pixels with readings from both sea ice and land ice cover. We grouped adjacent penguin colonies into three sub-regions (Fig. 1): (1) the South Shetland Islands, (2) the ’northeast-of-gap’ Antarctic Peninsula, and (3) the ’southwest-of-gap’ Antarctic Peninsula, which extends from the gap south to Adelaide Island but not beyond. For each sub-region, we included all monthly averaged sea-ice concentration data (1979–2021) falling within a 150 km radius of each penguin colony in during summer and spring periods, when penguins are constrained to foraging in the vicinity of the colony (January-March [Q1] and October-December [Q4]) and within a 500 km radius, following Iles et al.^43^ for the fall and winter period, when they are no longer tied to the breeding colony and forage further out to sea (April–June [Q2] and July–September [Q4]).

Linear models were used to fit annual and quarterly sea-ice concentration (SIC) in each Year (*i*) throughout the study period (1979–2018); we assumed region-specific intercepts (*β*_0 *j*_) and slopes (*β*_1 *j*_) as follows

$${\text{SIC}}_{ij} \sim N\left( {\mu_{ij} ,\sigma^{{2}} } \right)$$

$$\mu_{ij} = \beta_{0j} + \beta_{{{\text{1j}}}} * {\text{Year}}_{i}$$

Models were fit in a Bayesian framework using the R package "rjags"^44^, an interface to JAGS^45^, in the R statistical environment (R Core Team, 2019). We used three Markov Chain Monte Carlo (MCMC) sequences from the posterior distribution of each model parameter. Posterior distributions were derived using three chains with 5000 samples (after applying a thinning rate of 4 from each respective chain) following a "burn-in" period of 5500 draws (10,500 iterations for each chain). Model convergence was assessed using a visual analysis of the posterior chains, in addition to the use of the Gelman-Rubin convergence diagnostic^46^. Posterior analysis indicated that models unambiguously converged. Parameter estimate plots were generated using the "MCMCvis" package^47^, while other plots were generated using the "ggplot2" package^48^ in the R statistical environment. Posterior predictive checks of estimated parameters were calculated using the ppcheck function in the "jagsUI" package^49^.

## Results

### Adélie penguins

**Figure 3:**
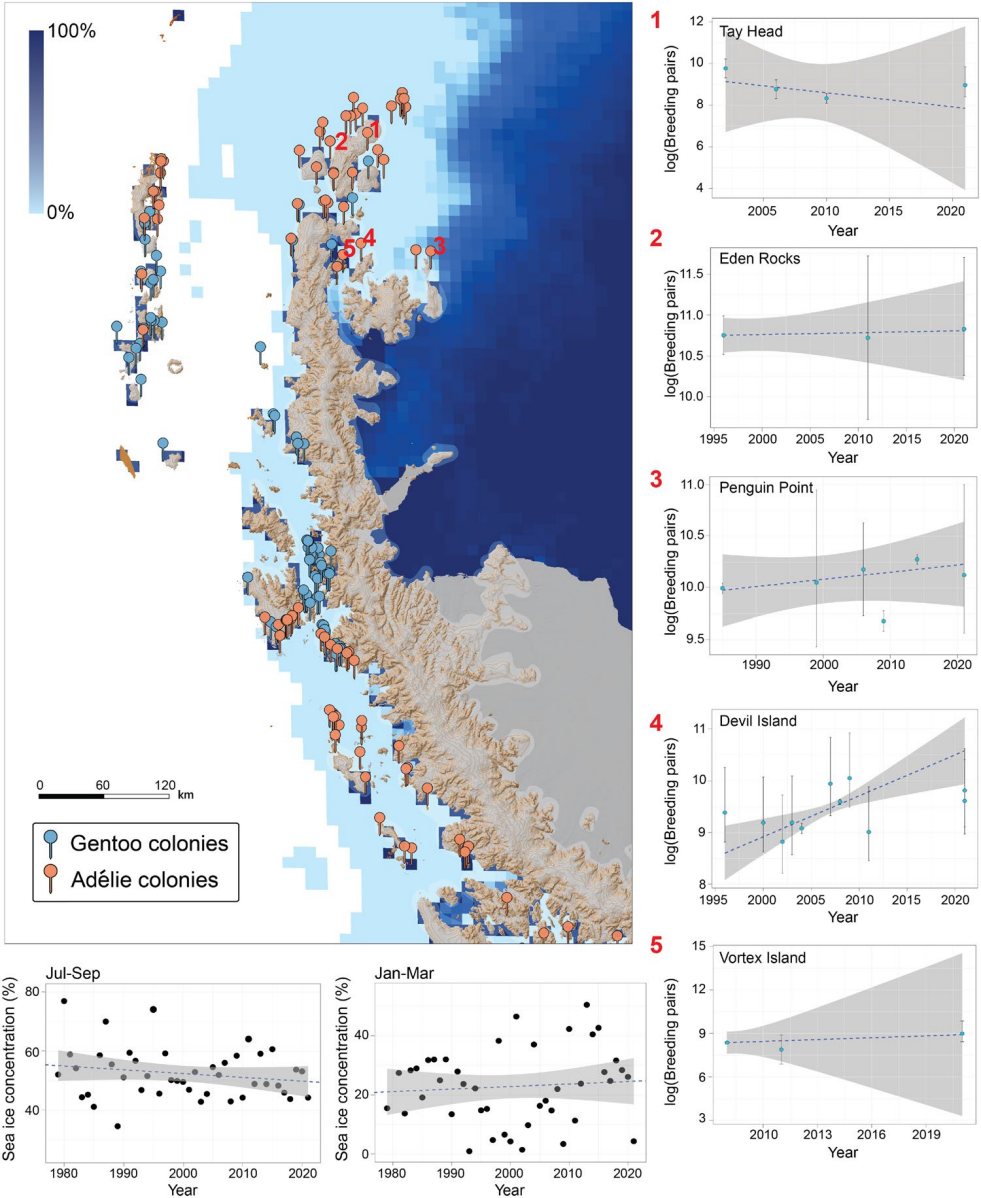
Population trends and mean annual sea-ice concentration trends for Adélie penguin colonies surveyed in the ’northeast-of-gap’ region. An example of summer [Q1] sea ice concentration is provided for illustration purposes, with blue-white scale ranging from 0 to 100% sea ice concentration. Figure created using ESRI ArcGIS Pro 3.0 (https://pro.arcgis.com) and Adobe Illustrator 27.0 (https://www.adobe.com/products/illustrator.html).

We surveyed nine Adélie penguin sites around the northern tip of the Antarctic Peninsula and into the Weddell Sea in January and February of 2022 (Fig. 3). At the time of our survey, penguin chicks had already begun créching so individual chick counts were performed rather than nest counts. In total, we counted 54,159 Adélie penguin chicks by hand across seven sites and 47,983 from RPAS imagery across two sites, for a total of 102,142 Adélie penguin chicks. One colony, Northeast of D’Urville (62.991 °S, 56.167 °W), was previously undocumented. Three colonies, Medley Rocks (62.997 °S, 56.023 °W), North of Medley Rocks (62.996 °S, 56.036 °W), and Cockburn Island (64.201 °S, 56.841 °W), were previously identified as penguin colonies from guano stains visible in NASA Landsat satellite imagery, but were of unknown species prior to this expedition^12^. Five sites (Devil Island, Vortex Island, Penguin Point, Eden Rocks, and Tay Head) had been previously surveyed at least once by a ground-based science team.

At Cockburn Island and Eden Rocks, surveyed solely by RPAS in this study, the total abundance was estimated by multiplying the total number of individuals (chicks and adults) visible at the site by an estimate of the fraction of all individuals that were chicks. At Cockburn Island, the fraction of chicks was estimated at 65% and our final estimate of the number of chicks at this site during the survey was 4274. At Eden Rocks, which was surveyed 2 days later, the fraction of chicks was 49%. Devil Island was surveyed by both RPAS and manual field counting, the latter of which provided a means of comparing the RPAS-derived estimate to the count completed in the field. At Devil Island, the chick fraction was 48%, yielding a chick estimate of 14,521. This RPAS-based estimate was 21% higher than the ground-based count of 11,978. We designated the RPAS counts as N3, meaning they are estimated to be within 10–25% of the true population size.

The number of breeding pairs on Devil Island grew at an annual rate of *r *= 0.08 (95% CI = [0.03, 0.12]) over the period from 1996 to 2021. Vortex Island, Eden Rocks, and Tay Head, and Penguin Point did not show significant trends in the number of breeding pairs over their survey histories (Vortex Island *r *= 0.04 [− 0.39, 0.48]; Eden Rocks *r *= 0.002 [− 0.02, 0.03]; Tay Head *r *= − 0.07 [− 0.37, 0.23]). See Table 1 for Adélie penguin census data for colony locations surveyed during this expedition. For details about statistical analyses of penguin colony population trends, please refer to Supplementary Materials B.

### Gentoo penguins

We counted 3694 gentoo penguin chicks across four breeding colonies. At the time of our survey, penguin chicks had already begun créching so individual chick counts were performed rather than nest counts. Two of the sites, Cape Scrymgeour and Northeast of D’Urville, had not previously been documented, and had 75 and 1917 chicks respectively. We identified the penguins previously noted (but not identified to species) at Medley Rocks (62.997 °S, 56.023 °W) and North of Medley Rocks (62.995 °S, 55.980 °W) as gentoo penguins with abundances of 1658 and 44 gentoo penguin chicks, respectively. We also confirmed the presence of breeding gentoo penguins (with chicks) at Wideopen Islands, a site where Adélie and chinstrap, but not gentoo, penguins^9^, were documented breeders. Unfortunately, we were not able to conduct a detailed survey to establish how many gentoo penguin pairs are currently breeding at this location. While gentoo penguins were the only penguin species breeding on Cape Scrymgeour, the southernmost Weddell Sea gentoo penguin colony surveyed during this expedition (63.566 °S), they co-occurred with Adélie penguins on Northeast of D’Urville, Medley Rocks, and North of Medley Rocks, and with Adélie and chinstrap penguins on Wideopen Islands. See Table 2 for gentoo penguin census data for colony locations surveyed during this expedition.

**Table 2:** Gentoo penguin colony survey history.

Site	Year	Count	Citation	Method
Cape Scrymgeour (SCRY)	2021	75 (C1)	This survey	Ground/RPAS
Medley Rocks 1 (part of MEDL)	2021	1658 (C1)	This survey	Ground
N. of Medley Rocks (NMED)	2021	44 (C1)	This survey	Ground
Northeast of d’Urville (NEDU)	2021	1917 (C1)	This survey	Ground/RPAS

Available gentoo penguin census data for colony locations surveyed during this expedition. Additional data from previous surveys have been included so as to place new data in its historical context; these data may also be downloaded from the Mapping Application for Penguin Populations and Projected Dynamics^7^ (MAPPPD; www.penguinmap.com). Each sites 4 letter MAPPPD code has been included. The methods of survey are: Ground, Survey was conducted by researchers on the ground; RPAS, Remotely-piloted aircraft systems; vhr, very-high resolution commercial satellite imagery; vessel, survey conducted by an offshore vessel.

### Confirmed absences

In addition to the surveys reported in Table 1, where penguins were found breeding, we surveyed four additional sites that had either appeared in historic accounts as having contained penguins^35^ or were identified in Landsat imagery as having guano-like stains that might reflect penguin colonies (Lynch and Schwaller, Unpublished data). Our expedition was able to confirm the absence of penguin colonies at the following locations: Correy Island, Beak Island, and Red Island. We were unable to locate a previously reported penguin colony on Dundee Island nearby Petrel Station, and though the exact location of the historical account is unclear, we do not believe the colony remains. We also confirmed that Rosamel Island and Cape Gordon on Vega Island, the latter of which was thought might contain breeding seabirds based on Landsat satellite imagery, have no breeding seabirds.

### Patterns of sea ice distribution and change across the Antarctic Peninsula

**Figure 4:**
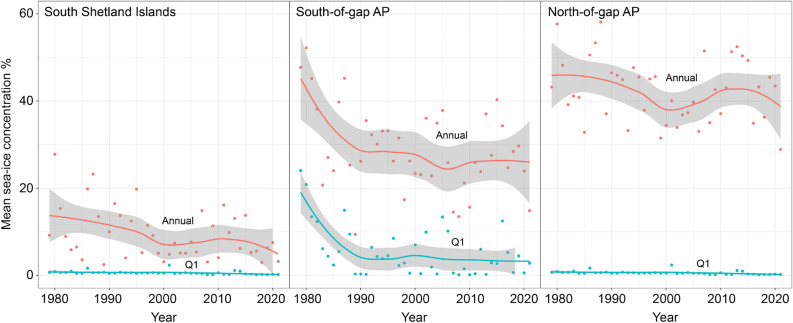
Mean annual and Q1 (January–March) sea-ice concentration trends 1979–2021. Red and green curves represent the smoothed regression model fit for annual and first quarter trends respectively, while the gray ribbons represent the 95% credible intervals.

We analyzed annual and quarterly patterns (Q1: January–March, Q2: April–June, Q3: July–September, Q4: October–December) of sea-ice concentration across the three regions: (1) South Shetland Islands, (2) ’northeast-of-gap’, and (3) ’southwest-of-gap’) from 1979 to 2021 (Fig. 4). For all regions, year-round average sea-ice concentrations declined from 1979 to 2021 and, with one notable exception, this average yearly sea ice decline was reflected in similar declines during each part of the year (Table 3). The South Shetland Islands region was characterized by declines in sea-ice concentration for all times of the year, though the declines in austral summer (Q1: January–March) fell short of being statistically significant. The coastline south of the Adélie gap exhibited significant declines in sea-ice concentration across all quarterly periods and were larger in magnitude than declines observed in either of the two regions further north. The sole exception to this overall loss of sea ice was in the austral summer (Q1; January–March) along the northern Antarctic Peninsula and Weddell Sea region, where sea-ice concentrations have increased slightly over the last four decades.

**Table 3:** Posterior means for for the period annual and quarterly (Q1: January-March, Q2: April-June, Q3: July-September, Q4: October-December) sea-ice concentration for the period 1979-2021.

Region	Period	Scale (km)	Sea ice change	Trend
Northeast-of-gap	Q1	150	0.085	Increase
Northeast-of-gap	Q2	500	− 0.11	Decline
Northeast-of-gap	Q3	500	− 0.13	Decline
Northeast-of-gap	Q4	150	− 0.20	Decline
South Shetland Islands	Q1	150	− 0.012	No change
South Shetland Islands	Q2	500	− 0.12	Decline
South Shetland Islands	Q3	500	− 0.16	Decline
South Shetland Islands	Q4	150	− 0.17	Decline
Southwest-of-gap	Q1	150	− 0.21	Decline
Southwest-of-gap	Q2	500	− 0.24	Decline
Southwest-of-gap	Q3	500	− 0.11	Decline
Southwest-of-gap	Q4	150	− 0.31	Decline
Northeast-of-gap	Annual	500	− 0.07	Decline
South Shetland Islands	Annual	500	− 0.09	Decline
Southwest-of-gap	Annual	500	− 0.17	Decline

Reported trends of study regions (e.g., Northeast-of-gap, South Shetland Islands, and Southwest-of-gap) were analyzed at 150 km and 500 km spatial scales to account for seasonal differences (150 km: spring and summer, 500 km: fall and winter) in penguin foraging ranges. Bayesian posterior means are reported as Sea ice change along with associated directional trend.

## Discussion

This survey expands the knowledge of Adélie and gentoo penguin abundance throughout the northern tip of the Antarctic Peninsula region and includes previously unsurveyed colony locations. Based on our survey estimates, we find that the Adélie penguin populations northeast and northwest of the so-called “Adélie gap” have remained stable or even increased over the last 30–40 years despite dramatic declines across colonies farther south along the western side of the Antarctic Peninsula. While Adélie and gentoo penguin habitat preferences are usually considered a zero-sum game in which the former climate change "loser" declines in step with increases in the latter—considered a climate change "winner"—we find that both species appear to be flourishing northeast and northwest of the Adélie gap. While previous studies have documented growing or stable gentoo populations at established colonies in this region (e.g., Brown Bluff, D’Urville Monument^7^), our expedition found a small gentoo colony at Cape Scrymgeour that appears to be a new colonization and, with the exception of a single nest reported from nearby Jade Point, the most southerly colony of this species on the Weddell Sea side of the Peninsula. Based on patterns of growth of new gentoo colonies^50^, we estimate that the Cape Scrymgeour population has been in existence at least 5 years and possibly longer absent continued immigration from other established populations. While gentoo penguin population expansions along their southern range margin on the western side have been well reported^29^, our findings provide further evidence of gentoo range expansion along the southern margins of their range east of the Antarctic Peninsula in the northern Weddell Sea.

The findings of this survey provide further support for existence of two separate and geographically distinct populations of Adélie penguins. The apparent asymmetry of Adélie penguin abundance observed north and east of the “Adélie gap” was initially hypothesized by Fraser et al.^1^. More recently, using stable isotope analyses, Polito et al.^51^ determined that populations on either side of the gap have distinct pre-breeding foraging distributions with the northeast-of-gap Adélie penguins foraging in the Weddell Sea prior to arrival at the breeding colony and those southwest-of-gap foraging in the Bellingshausen Sea over the same period. Our own findings suggest that those colonies existing in the northern AP region have remained broadly stable over the past several decades. Given the relatively similar terrestrial environment just north and south of the Adélie gap, we hypothesize that different foraging conditions in the overwinter period may have a disproportionate impact on penguin overwinter survival and that this, as opposed to breeding success at the colony, may be primarily responsible for the strikingly different population trajectories. As winter pack ice obligate species, Adélie penguins rely on consolidated ice floes for resting sites, while open water allows access to foraging areas. Previous studies of at-sea foraging by Adélie penguins have found that survival probabilities of Adélie penguins are influenced by winter sea ice extent and that Adélie penguin survival in the Ross Sea was greatest in years characterized by intermediate sea ice extent and lower during years characterized by either low or high sea ice extent^52^. High sea ice extent could reduce penguin access to food in the Ross Sea by restricting penguins northward and limiting access to productive hunting grounds^53^; similarly, low sea ice extent could negatively affect penguin survival by keeping them south of productive waters. Moreover, numerous studies have linked reduced sea ice coverage to declines in krill and Antarctic silverfish (*Pleuragramma antarctica*) populations, important prey species for Adélie penguins^54–58^, as sea ice provides spawning habitat, food resources and nursery regions for eggs and early life stages^59–61^. Chapman et al.^62^ showed that silverfish in Adélie penguin diet plays an important role in the survival of fledged chicks and La Mesa et al.^63,64^ found that decreased silverfish abundance in the southern WAP was correlated with decreases in sea ice. In this context, it is important to highlight the striking asymmetry in annual and quarterly sea ice trends. The magnitude of decline in sea-ice concentration observed across penguin colonies south of the Adélie gap was considerably larger in all but winter (July–September) relative to trends observed northeast-of-gap and in the South Shetland Islands. Along the WAP, substantial declines in Adélie penguin population abundance has been associated with multi-year and decadal scale trends of reduced lower sea-ice concentrations. While the logistical difficulties of tracking Adélie penguins during the overwinter period unavoidably limits our understanding of winter foraging behavior and its relationship to environmental conditions, it’s clear that sea ice can have both direct and indirect influences on winter foraging habitat with concomitant changes in the size of breeding colonies through time. While much of the discussion has focused on Adélie losses, our findings provide hope that where sea ice is being maintained, Adélie populations remain robust.

Despite the seemingly intuitive connection between sea ice and population trends, we recognize the challenge of suggesting any direct mechanistic relationship that such an analysis may provide in the absence of higher resolution datasets. The 25 km × 25 km spatial resolution of the passive microwave data used is likely inadequate to identify the precise mechanisms at play as ice-dependent wildlife interact with sea ice on an extremely localized basis. The characteristics of sea ice that might influence individual and group decisions about movement, foraging, or reproduction occur at scales far smaller than the resolution of most publicly available sea-ice data products. With this consideration in mind, we believe future investigations may benefit by incorporating newer satellite-based platforms such as the European Space Agency’s Sentinel-1 or NASA’s ICESat-2.

### Considerations for conservation

Previous work already identified the northern Weddell Sea as a penguin hotspot of significant conservation value^21,65,66^. With consideration to the large and apparently stable Adélie penguin populations in the northeast-of-gap region, as well as potential for further colonization by expanding gentoo penguins, the northeast-of-gap region deserves particular consideration in the design and implementation of conservation measures. Of particular relevance is the Domain 1 MPA planning process, which has already indicated the northeast-of-gap area as being of high conservation value^26–28^. Moreover, evidence for more stable sea ice dynamics Northeast-of-gap relative to the region south of the Adélie gap is consistent with the idea that this region, especially further around the tip of the Peninsula into the Weddell Sea, may act as a habitat refuge under future climate change that deserves particular protection from other, non climate-related, disturbances.

### RPAS survey logistics and lessons learned

The use of RPAS for penguin surveys is rapidly becoming a standard tool in Antarctic wildlife surveys, and while some of our surveys simply could not have been completed using manual counts alone (e.g., Eden Rocks), the unavoidable delay in survey work due to the Covid-19 pandemic created challenges in the use of RPAS-based imagery not shared by earlier expeditions we had undertaken in the same region in previous years^21^. In particular, the phenology of breeding at the time of the survey plays an important role in the ease with which survey images can be used to estimate abundance. Ideally, surveys should be completed during nest incubation, when each nest is attended by a single stationary adult. By contrast, our surveys occurred later in the season when the site was comprised of both adults and large chicks. Adults and chicks could be distinguished in some portions of the imagery, and while we were able to use this to estimate the percentage of all individuals that were chicks, a more accurate survey would have been achieved if we had been able to visit the study locations nearer to late December or early January.

## Supplementary Information

Supplementary Information.

## Data Availability

All data and files relating to penguin colony population trends and sea-ice concentration analyses are included with this published article as a Supplementary Information file. RPAS image mosaics, ArcGIS shapefile layers for penguin nests, and additional photographic data collected during this expedition may be obtained from the authors on request.
