# Supplementary material for: Adélie penguins north and east of the ‘Adélie gap’ continue to thrive in the face of dramatic declines elsewhere in the Antarctic Peninsula region
Source: Sci Rep. 2023 Feb 13;13:2525. doi: 10.1038/s41598-023-29465-4 (PMC9923640; doi:10.1038/s41598-023-29465-4)
Supplement: Supplementary file 1 — Supplementary Information. [file 41598_2023_29465_MOESM1_ESM.docx]

Supplementary Material for

**Adélie penguins north and east of the ‘Adélie gap’ continue to thrive in the face of dramatic declines elsewhere in the Antarctic Peninsula region**

Michael Wethington^1^*^,^*^∗^,Clare Flynn^1^, Alex Borowicz^1^*^,^*^2^, Heather J. Lynch^1^*^,^*^3^

1Department of Ecology and Evolution, Stony Brook University

2School of Marine and Atmospheric Sciences, Stony Brook University

3Institute for Advanced Computational Science, Stony Brook University

# A - Differentiating chicks from adults in UAV imagery


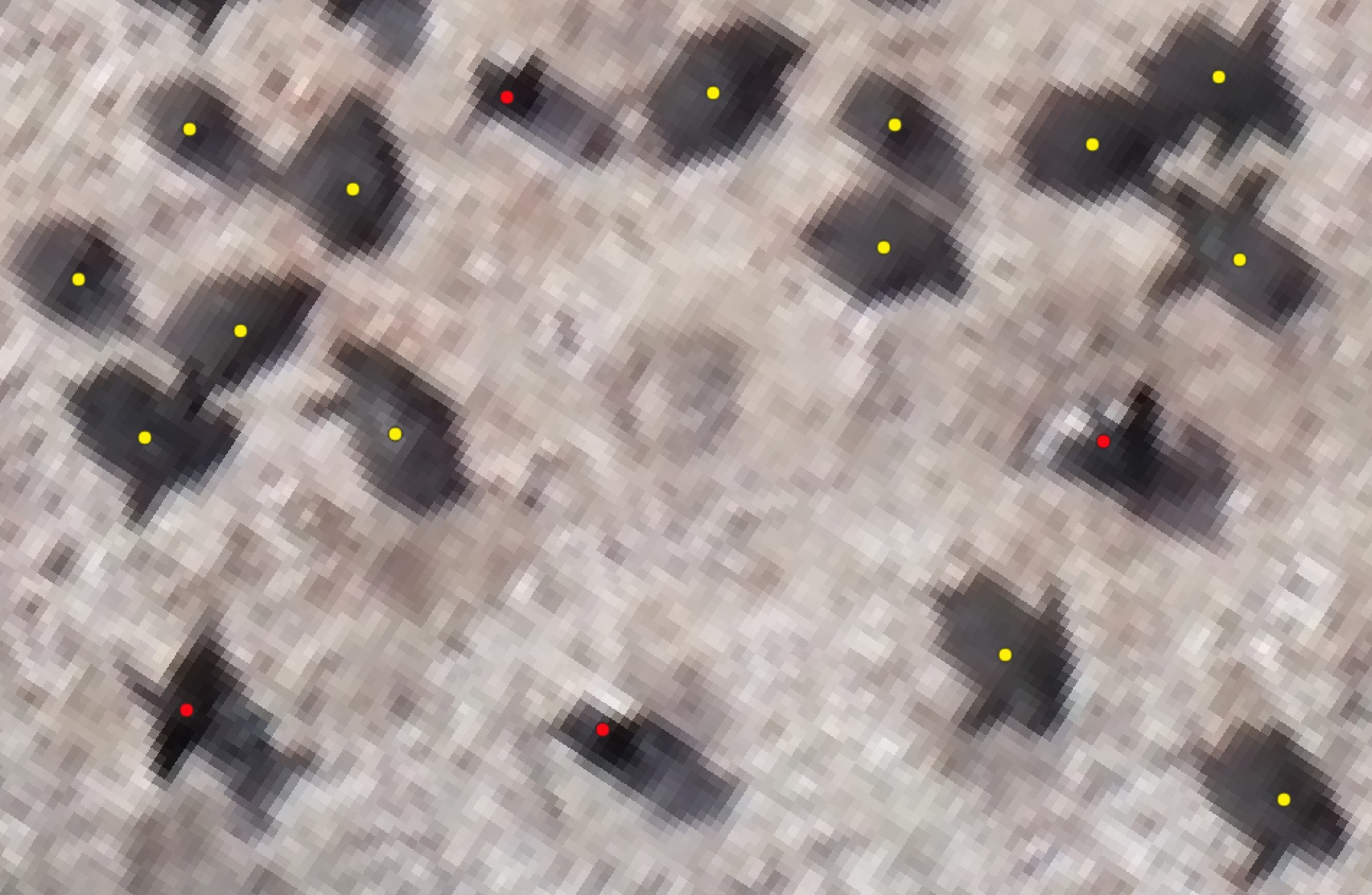


**Figure S1.** An image of Adélie penguins on Devil Island taken by RPAS with chicks marked by yellow dots and adults by red dots. Adults appear black with the occasional white belly visible and are sleeker and narrower than chicks at this stage of the season. Chicks appear gray, more rotund than the adults, and are often seen laying prone. Note that because chick down is quite fluffy, chicks often appear considerably larger than adults.

# B - Population Model

The population trends of the five sites with existing previous survey data were analyzed using weighted linear regression. Each population contains uncertainty due to observation error and, for chick counts converted to equivalent nest counts, the additional uncertainty associated with the number of chicks per nest. Our weighted linear regression weighted each point proportional to the inverse of the variance associated with each population estimate. Distributions of nest counts and chick counts were simulated from a log-normal distribution using the scale parameter *σ* based on the census accuracy level as described in Che-Castaldo et al.[^71^](#_bookmark68). In censuses where nest counts were conducted, the number of nests represents the number of breeding pairs at the colony. In censuses where chick counts were conducted because the chicks had reached the créche stage, the number of chicks was divided by the proportion of chicks that reach the créche stage in order to estimate the number of breeding pairs present at the colony. We doubled the créche success rate drawn from a Beta distribution with an expected value of 0.46 female chicks per nest as given by Hinke et al.[^72^](#_bookmark69). Because population estimates were log-transformed, the slope of the trend line represents the population growth rate.

In years with nest counts:

Breeding pairs ∼ *LogNormal*(*ln*(*NestCount*)*, σ* )

In years with chick counts:

Breeding pairs = Number of chicks*/*Reproductive success

where

Number of chicks ∼ *LogNormal*(*ln*(*ChickCount*)*, σ* )

and

Reproductive success ∼ 2 ∗ *Beta*(*α* = 4*.*6184*, β* = 5*.*4216)

where sigma is appropriate to the observation error (see Supplementary Materials 1 in Che-Castaldo et al.[^71^](#_bookmark68)).


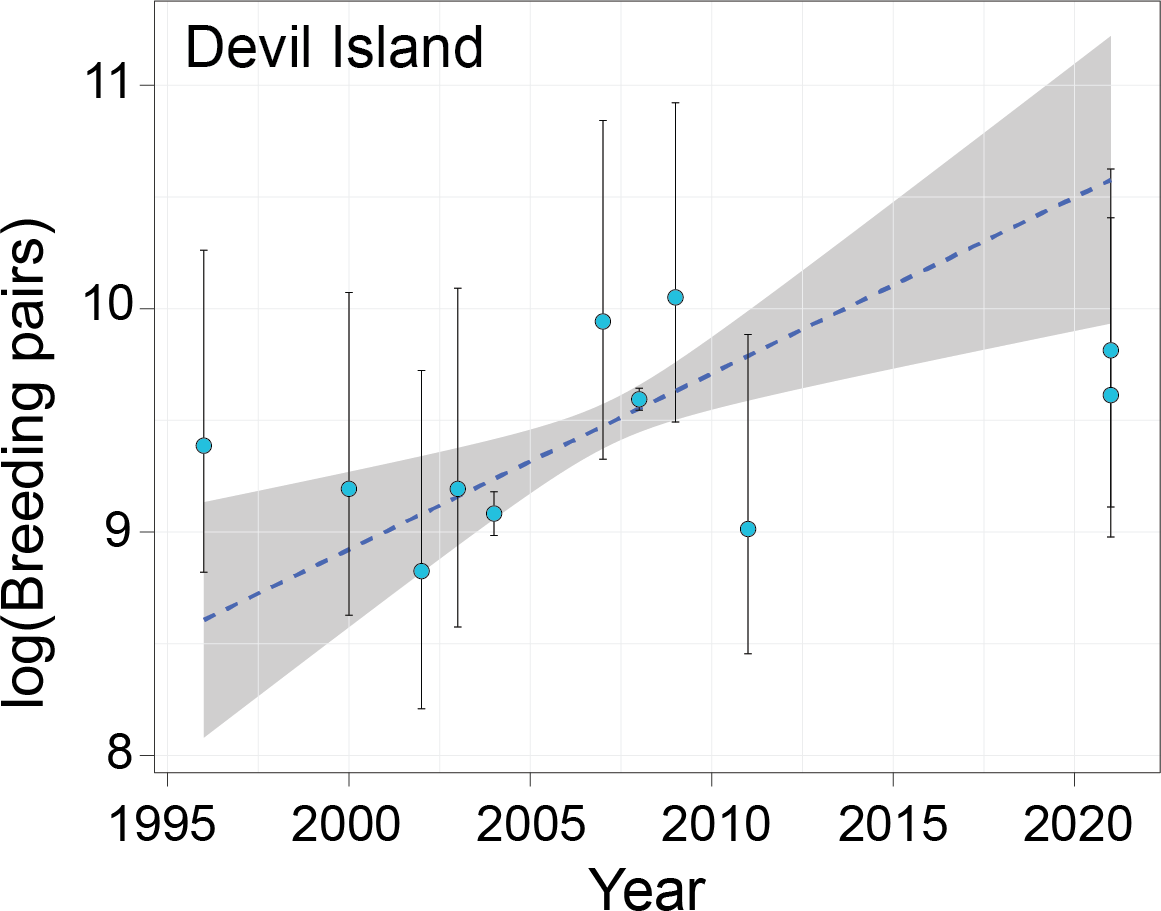


**Figure S2.** The natural log of the number of breeding Adélie penguin pairs on Devil Island since 1997. The error bars represent the 2.5^th^ and 97.5^th^ percentiles for each year, and the trend line is the weighted linear regression. Note that this plot is the same as shown in the panel to Figure 3; it is provided here in larger format for clarity.


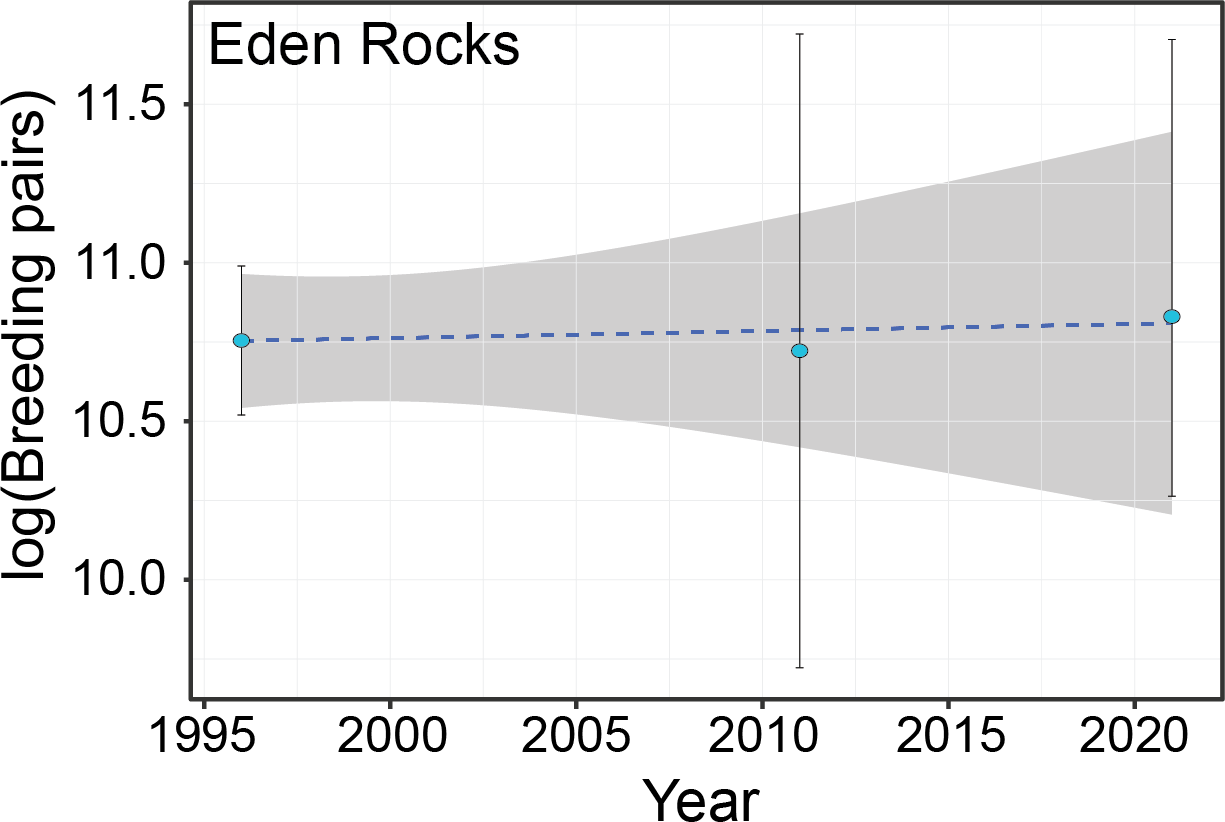


**Figure S3.** The natural log of the number of breeding Adélie penguin pairs on Eden Rocks since 1997. The error bars represent the 2.5^th^ and 97.5^th^ percentiles for each year, and the trend line is the weighted linear regression. Note that this plot is the same as shown in the panel to Figure 3; it is provided here in larger format for clarity.


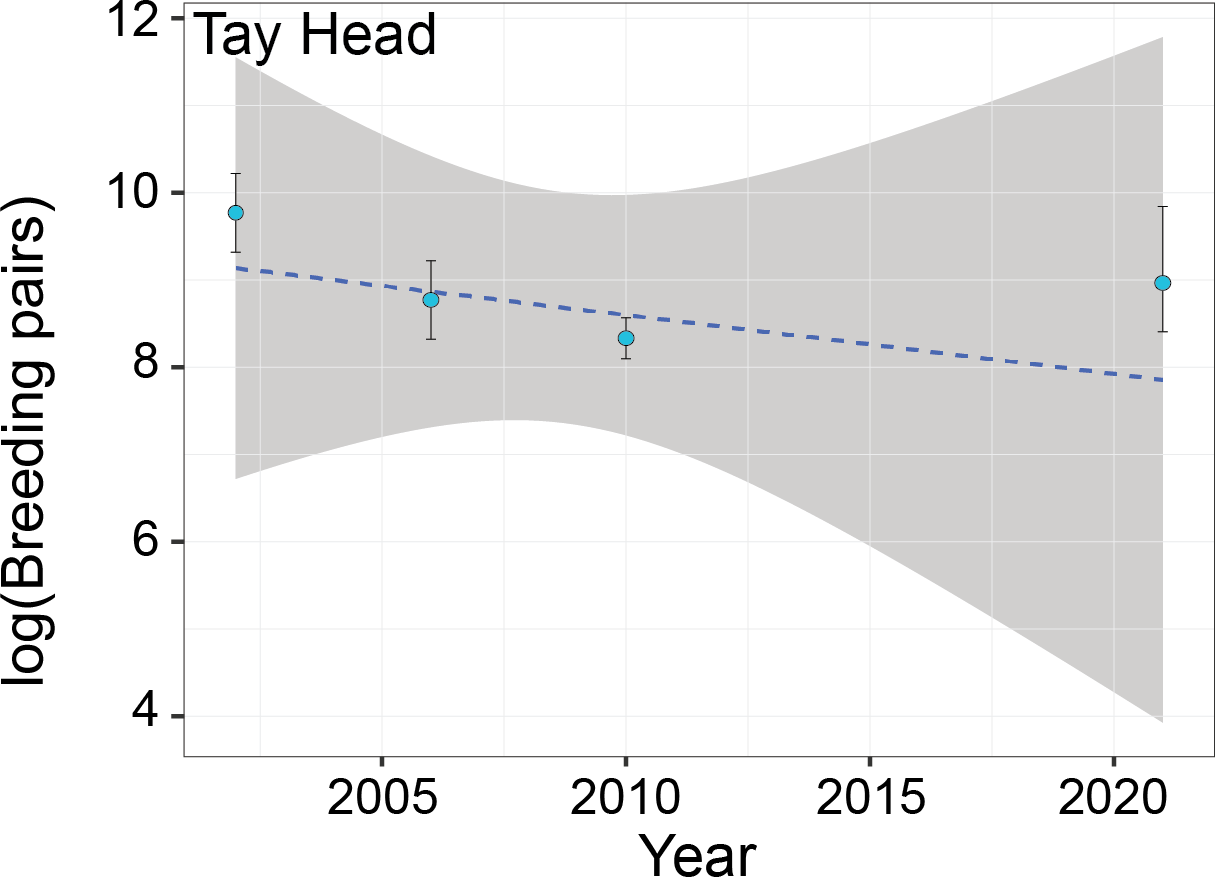


**Figure S4.** The natural log of the number of breeding Adélie penguin pairs on Tay Head since 2002. The error bars represent the 2.5^th^ and 97.5^th^ percentiles for each year, and the trend line is the weighted linear regression. Note that this plot is the same as shown in the panel to Figure 3; it is provided here in larger format for clarity.


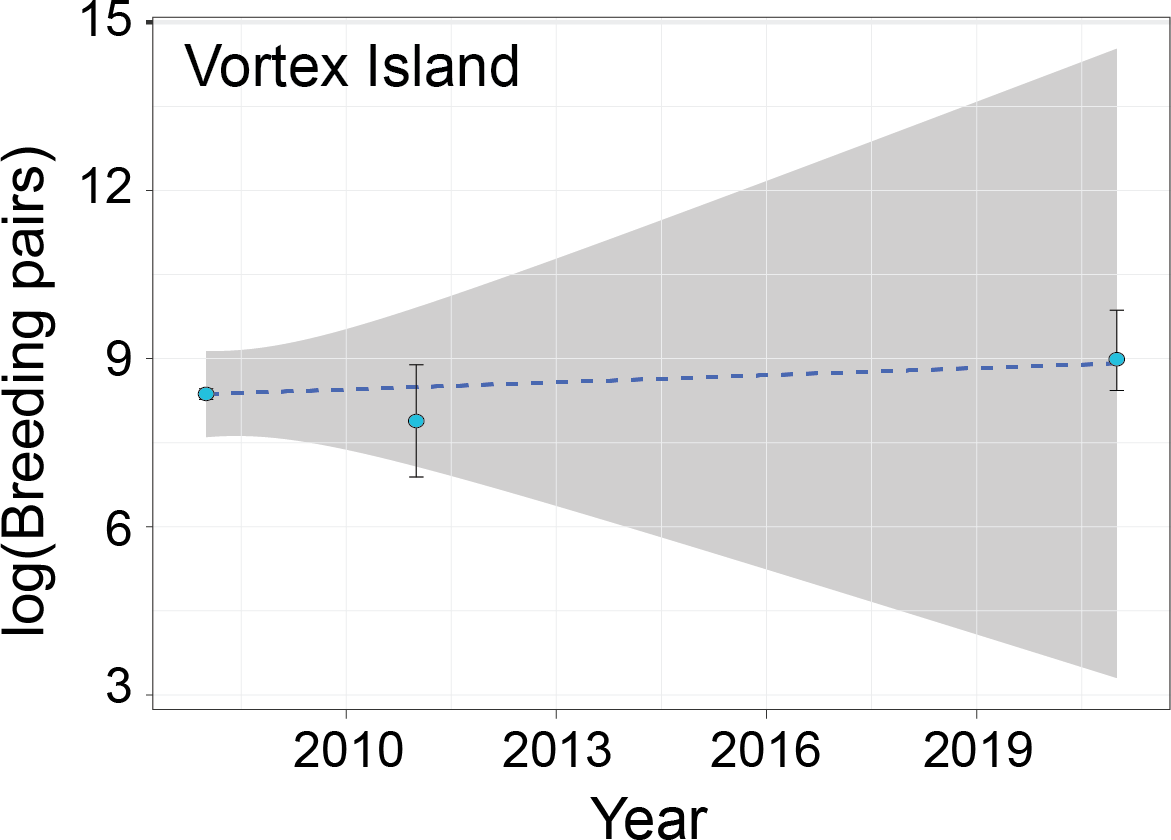


**Figure S5.** The natural log of the number of breeding Adélie penguin pairs on Vortex Island since 2008. The error bars represent the 2.5^th^ and 97.5^th^ percentiles for each year, and the trend line is the weighted linear regression. Note that this plot is the same as shown in the panel to Figure 3; it is provided here in larger format for clarity.


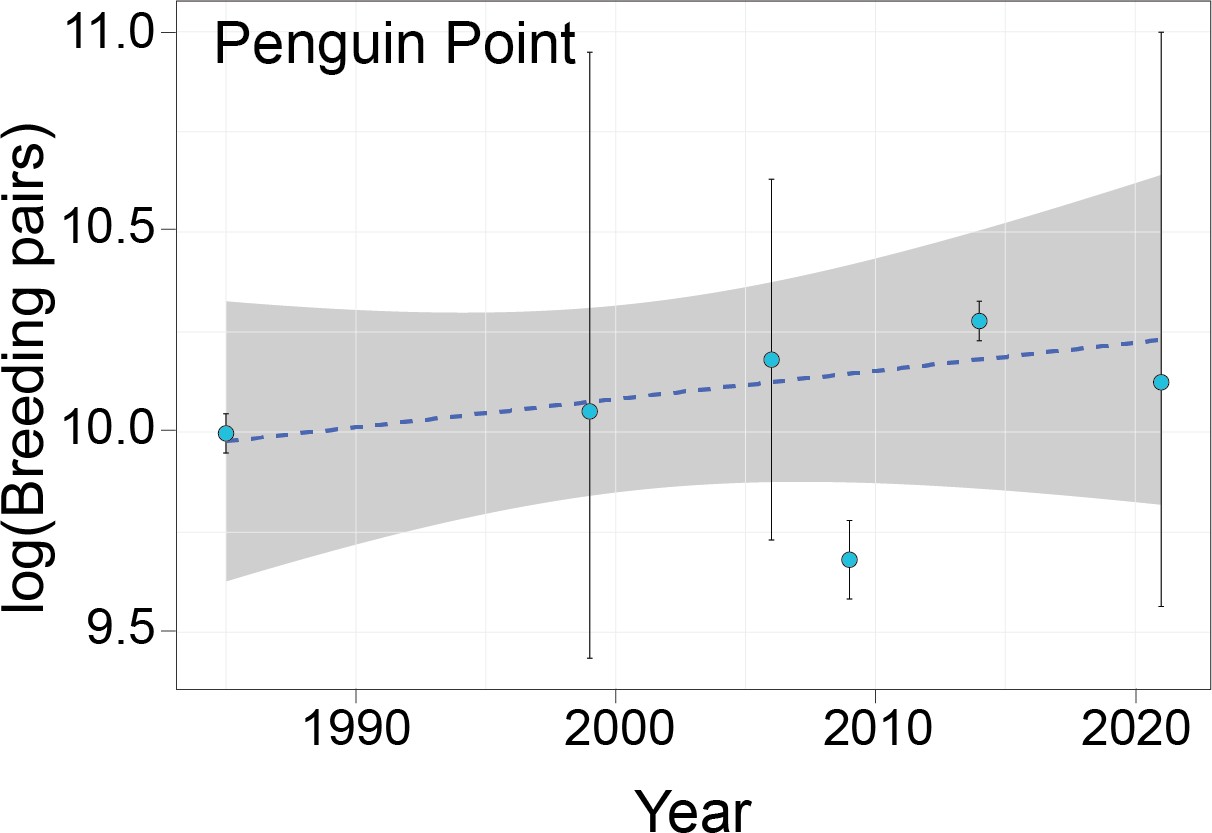


**Figure S6.** The natural log of the number of breeding Adélie penguin pairs on Penguin Point since 1985. The error bars represent the 2.5^th^ and 97.5^th^ percentiles for each year, and the trend line is the weighted linear regression. Note that this plot is the same as shown in the panel to Figure 3; it is provided here in larger format for clarity.
